# Emerging Perspectives on Gonadotropin Regulation in Vertebrates Revealed by the Discovery of FSH‐RH in Teleosts

**DOI:** 10.1002/bies.70066

**Published:** 2025-09-10

**Authors:** Daichi Kayo, Shun Kenny Uehara, Muhammad Rahmad Royan, Shinji Kanda

**Affiliations:** ^1^ Graduate School of Agriculture Kyoto University Kyoto Kyoto Japan; ^2^ Atmosphere and Ocean Research Institute The University of Tokyo Kashiwa Chiba Japan

**Keywords:** cholecystokinin, evolution, follicle‐stimulating hormone‐releasing hormone (FSH‐RH), gonadotropin‐releasing hormone (GnRH), hypothalamic–pituitary–gonadal (HPG) axis, reproduction, vertebrate

## Abstract

Vertebrate gonadal function is regulated by pituitary gonadotropins, follicle‐stimulating hormone (FSH) and luteinizing hormone (LH). These hormones are considered to be regulated by hypothalamic factor(s). Since the discovery of gonadotropin‐releasing hormone (GnRH) in mammals, which stimulates the secretion of both FSH and LH, GnRH had been believed to be the sole gonadotropin‐releasing hormone in vertebrates for more than 5 decades. However, recent studies have identified an alternative primary regulator of FSH in teleosts, leading to the hypothesis that FSH and LH are regulated by different factors in teleosts (dual GnRH model). This contrasts with the situation in mammals, where a single GnRH regulates both hormones (solo GnRH model). Importantly, although underlying mechanisms likely differ, both teleosts and mammals reproduce efficiently and have convergently evolved similar phenomena, including steroid feedback regulation. In this review, by comparing these taxa, we summarize mechanistic differences and propose an evolutionary scenario based on current experimental evidence.

## Graphical abstract

Regarding pituitary gonadotropin regulation, recent studies have shown that in mammals, FSH and LH are regulated by a single GnRH (solo GnRH model), whereas in teleosts, they are regulated by CCK (FSH‐RH) and GnRH (LH‐RH), respectively (dual GnRH model). This review outlines the mechanistic differences and suggests their evolutionary trajectories.

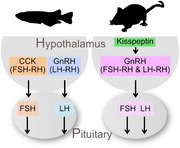

## 1 Introduction

Animals develop their gonads under appropriate conditions, such as their body size and environmental factors. Mechanistically, the regulation of the gonads is coordinated by the hypothalamic–pituitary–gonadal axis (HPG axis). Information from internal and external signals is integrated into the brain, and the hypophysiotropic neuropeptidergic neurons in the hypothalamus integrate these signals and stimulate hormone secretion from the pituitary gland. The pituitary hormones that stimulate gonadal development are referred to as gonadotropins, follicle‐stimulating hormone (FSH) and luteinizing hormone (LH), as they play exclusive roles in stimulating gonadal maturation through the general circulation. Once mature, the gonads produce sex steroid hormones, which endocrinologically act on the hypothalamus and/or pituitary, forming a feedback loop for the modulation of gonadotropin secretions. This reproductive control has been considered to be widely conserved across vertebrates, and its molecular mechanisms have been extensively studied in mammals.

However, accumulating body of evidence indicates some inconsistency in hypothalamic factors, such as the mode of gonadotropin release, between two well‐studied groups, teleosts and mammals. The canonical understanding established in mammals, in which hypothalamic gonadotropin‐releasing hormone (GnRH) is the sole regulator of FSH and LH, had long been considered applicable to vertebrates.

Recently, in teleosts, hypothalamic cholecystokinin (CCK), referred to as FSH‐releasing hormone (FSH‐RH), has been proposed as the primary releasing hormone for FSH, whereas GnRH primarily regulates LH secretion. This discovery revealed a structural difference in hypothalamic regulators between mammals and teleosts: Mammals utilize a single gonadotropin regulator (GnRH), whereas teleosts utilize two (conventional GnRH as LH‐RH and the novel “GnRH” as FSH‐RH). Importantly, despite each underlying mechanism being distinct, both species reproduce efficiently and exhibit similar secretory profiles of gonadotropin release, including sex steroid feedback regulation. In this review, by focusing on these two large taxonomic groups of vertebrates, we summarize the literature essential for understanding the commonalities and differences in their reproductive endocrine control.

## 2 Evolution of the Hypothalamic Regulatory Mechanism of the HPG Axis

### 2.1 GnRH is the Regulator of FSH and LH in Mammals, Whereas It Is an LH‐RH in Teleosts

The GnRH peptide was discovered in 1971 as the key regulator of FSH and LH release in mammals, in which Roger Guillemin and Andrew Schally won the Nobel Prize in Physiology or Medicine in 1977 for the significant breakthrough [1, 2]. In mammals, analysis of *Gnrh1*‐deficient mice revealed drastic reductions in the secretion of both FSH and LH, demonstrating that GnRH is the primary regulator of both hormones. In addition, since the administration of GnRH peptides induces both FSH and LH in many vertebrate species [2, 4, 5, 6, 7, 8, 9], the working hypothesis established in mammals had been assumed to be applicable to vertebrates in general. Therefore, GnRH neurons had been considered to constitute the final common pathway for regulating fertility in vertebrates in general [10, 11, 12].

Studies using teleosts have partially supported this hypothesis. Intraperitoneal injections of GnRH analogs have shown that GnRH is crucial for reproductive regulation in teleosts [7, 8, 13]. GnRH robustly increases blood LH levels, whereas blood FSH levels rise at relatively lower levels. Ca^2+^ imaging analysis of FSH cells and LH cells also revealed that GnRH peptides stimulate the release of both LH and FSH [14]. Later, the emergence of genome editing techniques made it possible to examine the essentiality of GnRH in regulating gonadotropin secretion in some teleost species. Interestingly, GnRH knockout (KO) females exhibited ovulation failure with significantly reduced *lhb* expression in the pituitary, whereas folliculogenesis and *fshb* expression were normal [15, 16]. These results indicate that while GnRH is essential for LH secretion, it is dispensable for FSH secretion in teleosts. Thus, although GnRH pharmacologically stimulates the secretion of FSH and LH, its physiological impact on FSH in teleosts appears to be different from that in mammals.

### 2.2 In Mammals, One GnRH Differentially Regulates Folliculogenesis and Ovulation via the Pituitary

Two modes of LH release have been found in mammals: pulse and surge. The LH pulse is found exclusively in mammals and plays a role in regulating folliculogenesis alongside FSH. On the other hand, the LH surge triggers final oocyte maturation and ovulation. GnRH not only regulates these multiple modes of LH but also the release of FSH, which contributes to both the proliferation of oocytes and the early process of folliculogenesis. Thus, how GnRH differentially regulates the release of FSH and the LH pulse/surge has long been questioned. In 2004, the kisspeptin neuronal system began to be proposed as a primary regulator of GnRH neurons [17, 18, 19, 20]. Two populations of kisspeptin neurons stimulate GnRH release in two ways [21]. First, the pulsatile release of GnRH/LH is regulated by kisspeptin neurons located in the arcuate nucleus (ARC) of the hypothalamus [22, 23]. These neurons co‐express neurokinin B (NKB) and dynorphin (Dyn), and are also known as KNDy neurons [24, 25, 26]. NKB facilitates their firing activity in an autocrine/paracrine manner, while Dyn suppresses it [27, 28]. These two autocrine/paracrine regulators produce intermittent bursting firing activities in this kisspeptin/KNDy neural population. The release of kisspeptin produces a pulsatile release of GnRH as it travels to the adenohypophysis via the hypophyseal portal vessel; this is the mechanism underlying the generation of pulsatile LH release from the pituitary [29].

Additionally, as the firing activity and Kiss1 expression of KNDy neurons are suppressed by ovarian estrogen, this population is considered a negative feedback center of gonadotropin release [21]. On the other hand, the LH surge, which is generated by the positive feedback action of estrogen, is regulated by another population of kisspeptin neurons located in the anteroventral periventricular nucleus (AVPV) [30]. This population stimulates GnRH neurons when the serum estrogen level increases [21].In addition to the regulatory mechanism involving kisspeptin neurons, it is important to note that kisspeptin (Kiss1) KO in mice results in reduced gonads caused by attenuated FSH and LH, a phenotype similar to that in GnRH‐deficient mice [31, 32, 33, 34]. Given these lines of evidence, the kisspeptin–GnRH–LH/FSH pathway is the central regulatory pathway of reproduction in mammals. However, this regulatory pathway is strongly suggested to be mammalian‐specific. Birds lack the kisspeptin gene [35, 36], and knocking out kisspeptin in teleosts does not disrupt gonadal functions [37, 38], although sex behavior shows some defects in reproduction [39]. Overall, kisspeptin regulates GnRH and ultimately FSH and LH release in mammals, whereas a different reproductive pathway has been suggested in other vertebrates. Additionally, as mentioned above, the pulsatile LH release driven by kisspeptin is specific to mammals, and the mechanism by which one GnRH differentially stimulates FSH and LH in nonmammalian species remains an open question.

### 2.3 In Teleosts, FSH‐RH/FSH Regulates Folliculogenesis, Whereas LH‐RH/LH Triggers Final Oocyte Maturation and Ovulation

Recently, several studies have revealed the elusive reproductive pathway of nonmammalian vertebrates in teleosts. As described earlier, in GnRH KO medaka (*Oryzias latipes*) [15] and zebrafish (*Danio rerio*) [16], the function of FSH remains unaffected in contrast to severe defects in LH expression/function. Therefore, a strong regulator of FSH in the hypothalamus might exist. In this context, from genetic, morphological, and physiological analyses, Uehara et al. demonstrated that hypothalamic cholecystokinin (CCK), acting on CCK receptors on FSH cells, functions as an essential key regulator of FSH secretion, serving as the FSH‐releasing hormone (FSH‐RH) [40]. Hollander‐Cohen et al. also conducted an important study demonstrating that a CCK receptor is expressed in FSH cells and plays a significant and essential role in FSH secretion in zebrafish [41]. Furthermore, the possible contribution of the CCK receptor to FSH regulation has already been reported in multiple teleost species. Cck2rb, the receptor of CCK, is expressed in the FSH cells of medaka, zebrafish [41], tilapia (*Oreochromis niloticus*) [41, 42], and Japanese eel (*Anguilla japonica*) [40]. Knocking out the receptors in medaka and zebrafish results in a severe reduction in the expression and function of FSH. Particularly in medaka, reintroducing the CCK receptor specific to FSH cells in *cck2rb* global knockout recovers the function of FSH, which supports the hypothesis that the intrinsic CCK directly acts on FSH cells. Furthermore, in vitro and in vivo experiments have shown that CCK peptide (CCK‐8s), an intrinsic agonist of Cck2rb, strongly promotes FSH expression and release in medaka and zebrafish [40, 41]. These findings suggest that Cck2rb on FSH cells plays an important role in the expression and release of FSH in teleosts.

**FIGURE 1:**
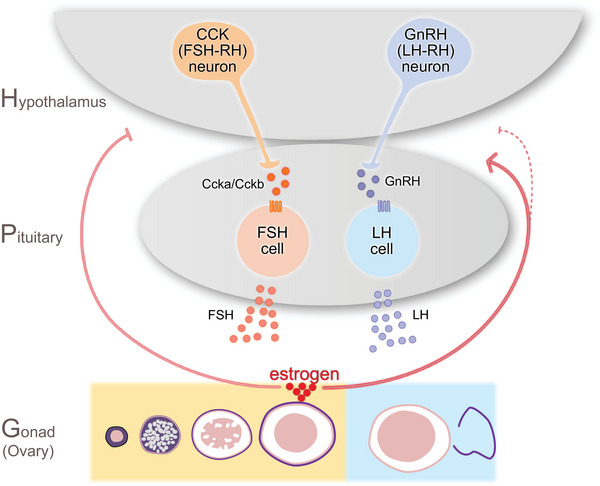
Working hypothesis of HPG axis regulation in female teleosts. FSH‐RH neurons in the hypothalamus induce folliculogenesis by stimulating FSH expression and secretion. Matured ovarian follicles secrete estrogen to suppress FSH secretion and to stimulate LH secretion, which induces final oocyte maturation and ovulation. This central regulatory mechanism can also be applied to males.

In contrast to the demonstration of the importance of the CCK receptor in FSH functions in multiple species, the demonstration of the intrinsic ligand and its neural system itself is limited in medaka. First, although Cck2rb can be activated by both gastrin and CCK, only the expression of CCK was detected in the hypothalamus of medaka, suggesting that CCK, but not gastrin, functions as an FSH‐RH [40]. Concerning the CCK genes, teleosts have two paralogs, namely, *cholecystokinin a* (*ccka*) and *cholecystokinin b* (*cckb*), which were identified in the teleost‐specific third‐round whole genome duplication (WGD) [40, 43]. In *ccka/b* double knockouts, FSH expression is reduced by more than 90%, which indicates that CCK functions as the primary regulator of FSH secretion. Additionally, histological analyses, including tracer experiments, have shown that hypothalamic CCK neurons in the nucleus ventralis tuberis (NVT) directly project to the pituitary, suggesting that the CCK in this population functions as an FSH‐RH (Figure 1). Since CCK‐immunoreactive cells are found in the hypothalamus, and fibers have been observed in the pituitary in other teleost species [41], the existence of hypophysiotropic CCK neurons in teleosts is highly likely. Taken together, it can be hypothesized that FSH‐RH is a general feature among teleosts. In addition to basic studies on its functions and relationships with other physiological states, investigations into its potential applications in aquaculture have just begun [44, 45, 46].

#### Nomenclature of the CCK receptors

In the nomenclature of the CCK receptor, there is a confusing situation. Vertebrates possess two types of CCK receptors, Cckar and Cckbr, based on mammalian nomenclature [47]. For the corresponding ligand genes in teleosts, CCKs are duplicated and are called *ccka* and *cckb* based on the rule that the products of teleost‐specific WGD are referred to as a and b [43]. Here, both gene products, Ccka and Cckb, can activate both teleost receptors, Cckar and Cckbr, which is inconsistent with what the nomenclature suggests. Therefore, we propose using the nomenclature Cck1r and Cck2r instead of Cckar and Cckbr to avoid misunderstanding.

## 3 Driving Force for the Evolution of Hypothalamic Gonadotropin Regulators in Vertebrates

These findings indicate that CCK primarily regulates FSH secretion, whereas GnRH primarily regulates LH secretion; this system is conserved among teleosts. This new model could be called as the “dual GnRH model” in contrast to the “solo GnRH model”, which represents GnRH as the primary regulator of both FSH and LH secretion in mammals. Although the evolutionary timing of the transition between the solo and dual GnRH models remains to be investigated, it is important to note that FSH and LH are expressed in the same cells in mammals but are expressed in separate cells in teleosts [2, 4, 5, 40, 41]. In the first‐ and second‐round WGD of the ancestral vertebrate [48, 49], the FSHβ and LHβ genes, the subunits of FSH and LH, were duplicated. Therefore, early vertebrates likely expressed FSH and LH in the same cells, which has persisted in mammals, leading to adaptive evolution constrained by the regulation of GnRH as a single regulator. On the other hand, teleosts evolved to have FSH and LH cells separately, which enabled them to receive two distinct regulators [49]. As sturgeon has been reported to exhibit separate expression of FSH and LH [50], this separation likely arose during the evolution of the actinopterygian lineage, prior to the divergence of teleosts. Generally, over the course of evolution, every duplicated gene pair tends to develop separate expression and becomes subject to distinct regulatory mechanisms, since differentiation can occur randomly and is irreversible (e.g., growth hormone, prolactin, and somatolactin [51, 52] and GnRH 1, 2, 3 [53]). Thus, it becomes natural to observe the dual GnRH model as a derived state in evolution in teleosts, whereas mammals retain aspects of the more ancestral solo GnRH model.

In addition to the separate expression of gonadotropins in terms of the cellular population, the functions of FSH and LH are clearly separate (FSH: folliculogenesis, LH: final oocyte maturation and ovulation) in female teleosts, which is different from the findings in mammalian females, whose LH is involved not only in ovulation but also in folliculogenesis [3, 54, 55, 56]. Knockout studies of gonadotropin genes in medaka and zebrafish indicate that FSH is essential for follicle development [15, 57, 58, 59], whereas LH triggers final oocyte maturation and subsequent ovulation [15, 58, 60], demonstrating a clear functional distinction between FSH and LH in teleost females. Thus, the dual GnRH system in teleosts aligns with the distinct expression and functions of FSH and LH, which may be a straightforward consequence of the sub‐functionalization of the duplicated genes.

In contrast, mammals exhibit a more complex situation, as a single cell secretes two gonadotropins under the regulation of two distinct control systems. The GnRH receptor (GnRHR) possesses a unique structural feature among G protein‐coupled receptors (GPCRs). This protein is truncated at the C‐terminus, which results in impaired receptor functions under artificial prolonged exposure to GnRH [61, 62, 63], as the truncated C‐terminal tail is essential for binding to β‐arrestins, which mediate receptor internalization [62].

This may simply reflect the physiological context in mammals, where GnRH‐LH secretion is characterized by distinct pulse (generated by ARC‐KNDy neurons) and surge modes (by AVPV‐Kiss1 neurons); thus, situations involving prolonged GnRH exposure do not naturally occur in vivo. Recently, a transgenic mouse in which the C‐terminal tail of the chicken GnRHR gene was fused to the endogenous GnRHR gene was generated, and the pattern of LH secretion was examined [64]. While some impairments in fertility and blunted LH surges were observed, neither function was completely abolished. These findings support the above idea that the structural changes in the mammalian GnRHR C‐terminal tail may result from the lack of continuous exposure to GnRH caused by the pulsatile and surge mode of secretion rather than being purposefully acquired.

Thus, mammals and teleosts acquired their current different regulatory systems that adequately and essentially regulate their co‐expressed or differentially expressed gonadotropins, which can ultimately give rise to distinct models (solo GnRH vs. dual GnRH) of reproductive control.

## 4 Feedback Regulation of Gonadotropin Secretion in Both Models

The secretion of gonadotropins is stimulated when the gonads are immature and is subsequently suppressed by gonadal factors via a feedback mechanism as the gonads develop. This phenomenon has been reported in a wide range of vertebrate species, from fish to tetrapods [49, 65, 66, 67, 68, 69], highlighting its biological significance. The key mediators that provide feedback of the gonadal state on the hypothalamic regulators or gonadotrophs in the pituitary are sex steroid hormones. However, the precise site of action for this feedback regulation remains unknown in most vertebrates except mammals.

In mammals, Kiss1 neurons express estrogen receptor (*Esr1*), which mediates estrogen feedback signals [21] and regulates GnRH neurons. As described above, Kiss1 neurons in the ARC (KNDy neurons) contribute to the LH pulse (folliculogenesis), and those in the AVPV contribute to the LH surge. These two distinct Kiss1 neuron populations receive estrogen feedback; estrogen suppresses Kiss1 expression in the ARC but enhances it in the AVPV, corresponding to negative feedback during the LH pulse and positive feedback during the LH surge [70].

In teleosts, gonadotropin secretion is strongly influenced by sex steroid hormones. Although LH secretion is basically promoted by sex steroid hormones in many cases [71, 72, 73, 74, 75], some studies have reported a reduction in LH levels [76, 77]. Thus, excitatory and/or inhibitory inputs may contribute to the feedback mechanism regulating GnRH‐LH secretion [76, 77, 78, 79]. In contrast, FSH secretion is consistently downregulated by sex steroid hormones in various species: rainbow trout [71], coho salmon [72], goldfish [73], tilapia [80], sea bass [74], Atlantic croaker [75], medaka [49], and three‐spined sticklebacks [81], except in an in vitro analysis using pituitary cells in eel [82]. Therefore, a common primary mechanism for the negative feedback control of FSH production in teleosts can be expected.

In mammals, folliculogenesis and ovulation are controlled by either the GnRH pulsatile or surge secretion. Therefore, it is reasonable that the site of action for estrogen feedback is located in kisspeptin neurons, which regulate GnRH secretion patterns. On the other hand, in teleosts, the secretion of gonadotropins (FSH and LH) is controlled by FSH‐RH and LH‐RH, respectively. Assuming a similar mechanism, the site of sex steroid hormone feedback can be hypothesized to be located at the level of each stimulator, FSH‐RH and GnRH (LH‐RH) neuron. In fact, in medaka, it has been shown that LH‐RH neurons express Esr2a and exhibit an estrogen‐dependent increase in firing activity, suggesting that these neurons may directly receive estrogen feedback [83].

Despite consistent observations of negative sex steroid feedback across species [49, 71, 72, 73, 74, 75, 80, 81], the mechanism and site of FSH secretion in teleosts remain only partially understood. In medaka, it has been demonstrated that Esr2a KO females exhibit a significant increase in *fshb* expression. Moreover, the suppressive effect of estradiol‐17β (E2) administration on *fshb* expression was impaired in Esr2a KO females. Organ culture of isolated pituitaries revealed the existence of a direct suppression pathway of *fshb* expression by E2, which is also compromised in Esr2a KO females [84]. In goldfish, the expression of follistatin, which is known as activin‐binding protein, increases in a sex steroid‐dependent manner and suppresses FSH synthesis in the pituitary [85]. In addition to estrogen, inhibin, which has been identified as a direct suppressor of FSH secretion in mammals [86], is also suggested to suppress *fshb* expression in teleosts. Increased expression of *fshb* has been observed in *inhbaa/inhibab* double‐KO female zebrafish [87]. These findings suggest the existence of a direct negative feedback pathway from the gonads acting on FSH at the pituitary level. On the other hand, with the discovery of FSH‐RH neurons, which are hypothalamic regulators, their potential as another strong candidate for the site of action of estrogen negative feedback has been raised. Future studies are expected to uncover the mechanisms of gonadotropin secretion feedback regulation in the dual GnRH model.

## 5 Other Possible Direct and Indirect Regulators of Gonadotropin Secretion

The regulation of gonadotropins in vertebrates, including mammals and teleosts, may involve complex networks of hypothalamic neuropeptides, neurotransmitters, and modulatory factors in addition to the FSH‐RH and GnRH pathways. While GnRH and FSH‐RH are central to gonadotropin regulation, many previous studies have suggested potential alternative or redundant mechanisms enabling gonadotropin regulation and reproduction. Over the past 5 decades, research has increasingly revealed the complexity of the reproductive axis, in addition to what we currently understand from the kisspeptin–GnRH–FSH/LH (mammals) or FSH‐RH/GnRH–FSH/LH (teleost) paradigm.

### 5.1 Vasoactive Intestinal Peptide (VIP)

Approximately, 20–30 excitatory and inhibitory neuropeptides and neurotransmitters regulate GnRH and gonadotropins release in mammals and fishes, and have been reported to have some direct or indirect effects. For comprehensive reviews, we refer readers to Spergel [88] for mammals and Trudeau [77] for teleosts, while this review emphasizes key examples. Among them, vasoactive intestinal peptide (VIP) has emerged as a potent GnRH regulator. In mammals, VIP neurons in the suprachiasmatic nucleus (SCN) innervate GnRH neurons, coordinating the LH surge for ovulation [89]. Disruption of VIP or its receptor, VIP receptor type 2 (VPAC2), signaling in mice leads to estrous cycle irregularities and ovulatory deficits [90, 91], whereas the VIP peptide directly increases GnRH neuron firing and intracellular Ca^2+^ levels [92]. Although Vip has been reported to indirectly stimulate LH release independent of GnRH and dopamine in teleosts [93], its loss does not impair female reproduction but instead causes male subfertility [94]. Intriguingly, it has also been reported that gene knockout of neuropeptide FF (NPFF) causes subfertility specific to males in medaka [95]. While male gonadotropins and/or receptor KO teleosts present relatively mild or no obvious phenotypes, females present severe phenotypes, such as immature gonads or anovulation, that ultimately cause infertility [15, 57, 58, 59, 60], which is not consistent with the results of the knockouts of *npff*. Therefore, these male‐specific impairments may suggest the existence of other potential sites of action of the bioactive peptides in the regulation of gonadal maturation, in addition to stimulating GnRH or gonadotropin release. Additionally, while the loss‐of‐function experiments are thus far limited to model teleosts (mostly zebrafish and medaka), comparative studies, especially those involving anatomical and genetic studies, are needed to explore the evolutionary role of these factors among teleost species.

In addition to Vip, several stimulatory neuropeptides for gonadotropin release (primarily LH) in teleosts have been reported, including secretoneurin [96], galanin [97], pituitary adenylate cyclase‐activating polypeptide (PACAP) [98], and isotocin [99]. While exposure experiments suggest their potential regulatory effects on gonadotropins, further evidence from multiple studies on their loss of function via knockout is needed to confirm their strong contribution to gonadotropin synthesis and/or release. For example, secretogranin‐2 (*scg2*, the prohormone of secretoneurin) KO has been shown to reduce ovulation in females and courtship behaviors in male zebrafish, with reduced *gnrh3*, *fshb*, and *lhb* expression [100]. Although the results are striking, further functional analysis specific to the peptides or their unidentified receptor is needed to elucidate their role, as secretogranin‐2, which is produced from the same precursor as secretoneurin, is known to be involved in the packaging and sorting of peptide hormones and neuropeptides.

### 5.2 Thyroid‐Stimulating Hormone

Thyroid‐stimulating hormone (TSH) also exhibits species‐specific differences in its role in reproductive regulation. Seasonal breeders rely on photoperiodic cues to regulate reproduction, with TSH emerging as a key upstream regulator of GnRH. In mammals, melatonin from the pineal gland modulates pituitary *pars tuberalis* (PT)‐TSH, which acts in a retrograde manner on tanycytes to convert thyroxine (T4) into bioactive triiodothyronine (T3), ultimately reshaping the glia‒GnRH interaction that enables GnRH release and thus triggers LH release [101, 102, 103]. Until recently, this pathway has been widely accepted as the mechanism by which seasonal changes regulate reproduction in seasonal breeders, particularly in birds [101, 103]. However, no knockout studies have tested this hypothesis, as PT‐TSH and PD‐TSH differ only in glycosylation post‐translation [104]; thus, disrupting the TSH gene could severely impact metabolism and development. In teleosts, evidence suggests that TSH also regulates reproduction, with studies in medaka [105, 106], sticklebacks [107], and chub mackerel [108] linking *tshba* expression to gonadotropin expression and reproduction. Interestingly, in female medaka, TSH may bypass GnRH and act directly on gonadotrophs via folliculostellate cells [106]. Therefore, knockout studies in teleosts could help clarify the role of TSH in reproduction triggered by photoperiodic changes. Although knocking out *tshba* might be detrimental, teleosts still possess a paralog of this gene (i.e., *tshbb*) that offers a target for disruption. Indeed, studies in Atlantic salmon [109, 110] and medaka [106] suggest that the photoperiod responsiveness of *tshbb* expression might be associated with reproduction, but further studies including analyses of knockouts are needed to confirm this. Several questions remain whether the regulatory mechanisms of *tshbb* are the same as those of the canonical PT‐TSH pathway in other vertebrate species or whether they are the same among teleost species and even between sexes. Indeed, unlike in mammals, where PT‐TSH has thus far been established as a photoperiodic control of reproduction, its necessity in vertebrate reproduction remains uncertain and requires further investigation using teleosts as potential models.

## 6 Conclusions and Prospects

**FIGURE 2:**
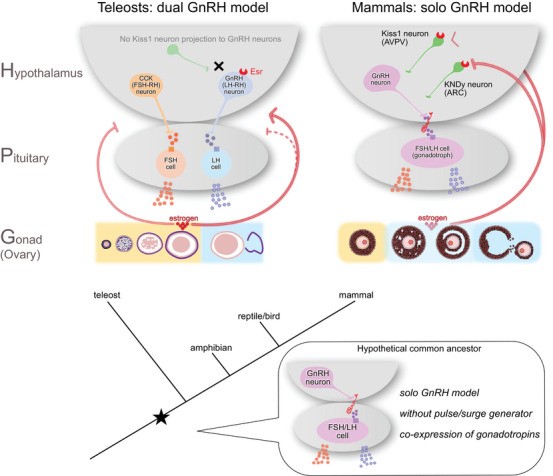
Solo GnRH model and dual GnRH model. A schematic illustration of the primary factors and mechanisms regulating the HPG axis in teleosts and mammals. We summarize the differences in hypothalamic regulators, the population of gonadotrophs, and the functions of gonadotropins. Although they have different regulators, they have convergently acquired precise control mechanisms of ovarian functions, including steroid feedback. Given the commonalities and differences between them, a hypothetical common ancestor is proposed in the figure; it possesses a solo GnRH model, without a pulse/surge generator, and shows co‐expression of FSH and LH.

Here, we summarize studies of the hypothalamic regulation of gonadotropins in mammals and teleosts and discuss possible evolutionary scenarios of the regulation of gonadotropin secretion. Teleosts have differentiated gonadotrophs into two distinct types, FSH cells and LH cells, and have also evolutionarily acquired two hypothalamic regulators, FSH‐RH and GnRH (LH‐RH). In contrast, mammals utilize a single hypothalamic regulator, GnRH, but acquire ARC or AVPV kisspeptin neurons as upstream regulatory mechanisms (Figure 2, right panel). It should be noted that these two strategies were independently evolved from the same ancestral regulatory system. Given the experimental evidence so far, we propose a hypothetical common ancestor in the figure (Figure 2, lower panel). Further investigations of basal vertebrates (cyclostomes, cartilaginous fishes, and basal bony fishes) may provide a more solid hypothesis regarding this ancestral system. The understanding of this evolutionary trajectory will provide important biological insights not only into reproductive endocrinology but also into the broader understanding of the functional and expressional differentiation of paralogs after gene duplication.

To date, many regulators and factors involved in gonadotropin secretion have been identified in teleosts. Among them, distinguishing which ones are the primary regulators and which ones serve as auxiliary regulators is essential. With the discovery of the dual GnRH model in teleosts, accumulating evidence suggests that GnRH serves as the primary regulator of LH secretion, whereas FSH‐RH (identified as CCK) is the primary regulator of FSH secretion. By adopting the dual GnRH model, teleosts can regulate folliculogenesis and ovulation independently, which could offer an adaptive advantage in their reproductive strategies.

Moreover, although this review has not addressed basal vertebrates, amphibians, reptiles, or birds, future studies elucidating their respective gonadotropin regulatory models and hypothalamic regulators will help clarify how the HPG axis has evolved across all vertebrates to reach its current regulatory system.

## Author Contributions

D.K. wrote the draft, assembled the drafts from all authors, edited and revised the manuscript, and helped with supervision and finalization. S.U. wrote the draft and revised the manuscript. M.R. wrote the draft and revised the manuscript. S.K. conceptualized and supervised the work, edited, revised, and finalized the manuscript.

## Conflicts of Interest

S.K. has filed a patent related to this study (Japanese Patent Application No. 2023–25897). The remaining authors declare no competing interests.

## Data Availability

Data sharing is not applicable to this article as no datasets were generated or analyzed during the current study.
