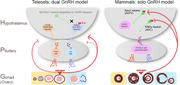# Erratum "Emerging Perspectives on Gonadotropin Regulation in Vertebrates Revealed by the Discovery of FSH‐RH in Teleosts"

**DOI:** 10.1002/bies.70111

**Published:** 2026-02-01

D. Kayo, S.K. Uehara, M.R. Royan, and S. Kanda, “Emerging Perspectives on Gonadotropin Regulation in Vertebrates Revealed by the Discovery of FSH‐RH in Teleosts,” *Bioessays* 47 (2025): e70066. http://doi.org/10.1002/bies.70066.

In the article published as Bioessays 2025; 47: 70066, https://doi.org/10.1002/bies.70066 the figure 2 should be updated, as one arrow was removed during the production process and was overlooked in proofreading. The correct figure statement is: